# Calcium Channels in Adult Brain Neural Stem Cells and in Glioblastoma Stem Cells

**DOI:** 10.3389/fncel.2020.600018

**Published:** 2020-11-13

**Authors:** Valérie Coronas, Elodie Terrié, Nadine Déliot, Patricia Arnault, Bruno Constantin

**Affiliations:** Laboratoire STIM, Université de Poitiers-CNRS ERL 7003, Poitiers, France

**Keywords:** store-operated channel, calcium toolkit, neural stem cells, brain, glioma, glioblastoma, cancer stem cell, calcium channel

## Abstract

The brain of adult mammals, including humans, contains neural stem cells (NSCs) located within specific niches of which the ventricular-subventricular zone (V-SVZ) is the largest one. Under physiological conditions, NSCs proliferate, self-renew and produce new neurons and glial cells. Several recent studies established that oncogenic mutations in adult NSCs of the V-SVZ are responsible for the emergence of malignant primary brain tumors called glioblastoma. These aggressive tumors contain a small subpopulation of cells, the glioblastoma stem cells (GSCs), that are endowed with proliferative and self-renewal abilities like NSCs from which they may arise. GSCs are thus considered as the cells that initiate and sustain tumor growth and, because of their resistance to current treatments, provoke tumor relapse. A growing body of studies supports that Ca^2+^ signaling controls a variety of processes in NSCs and GSCs. Ca^2+^ is a ubiquitous second messenger whose fluctuations of its intracellular concentrations are handled by channels, pumps, exchangers, and Ca^2+^ binding proteins. The concerted action of the Ca^2+^ toolkit components encodes specific Ca^2+^ signals with defined spatio-temporal characteristics that determine the cellular responses. In this review, after a general overview of the adult brain NSCs and GSCs, we focus on the multiple roles of the Ca^2+^ toolkit in NSCs and discuss how GSCs hijack these mechanisms to promote tumor growth. Extensive knowledge of the role of the Ca^2+^ toolkit in the management of essential functions in healthy and pathological stem cells of the adult brain should help to identify promising targets for clinical applications.

## Introduction

In the brain of adult mammals, including humans, neural stem cells (NSCs) reside within two major regions: the ventricular-subventricular zone (V-SVZ, also called the subependymal zone or subventricular zone) lining the lateral brain ventricles and the subgranular zone of the dentate gyrus in the hippocampus. Within these niches, NSCs sustain lifelong neurogenesis and gliogenesis (Lim and Alvarez-Buylla, 2016; Lledo and Valley, 2016; Obernier and Alvarez-Buylla, 2019). In this review, we focus primarily on the V-SVZ that is the largest germinal region of the adult brain. Within this neurogenic area, quiescent and activated NSCs coexist. Upon activation, NSCs divide to produce transient amplifying progenitors that engender neuroblasts that in turn, replenish the olfactory bulb population of interneurons. Adult NSCs in the V-SVZ also generate astrocytes and oligodendrocytes that disperse within the brain parenchyma (Lim and Alvarez-Buylla, 2016; Lledo and Valley, 2016; Obernier and Alvarez-Buylla, 2019).

While NSCs are mobilized by brain injuries and contribute to attempts of brain repair, recent studies have consistently established that oncogenic mutations in NSCs from the V-SVZ, unfortunately, lead to the development of glioblastoma, one of the deadliest cancers in adults (Recht et al., 2003; Barami, 2007; Quiñones-Hinojosa and Chaichana, 2007; Lee et al., 2018). These malignant tissues contain a subpopulation of cells, called glioblastoma stem cells (GSCs) that share several properties with NSCs from which they may derive (Recht et al., 2003; Zarco et al., 2019). Because of their growth properties and ability to resist the current treatments, GSCs are considered responsible for tumor initiation, growth, and relapse (Galli et al., 2004; Singh et al., 2004).

NSCs and GSCs are controlled by multiple extracellular signals, numbers of which recruit Ca^2+^ signaling actors to transduce their effects. Ca^2+^ is a ubiquitous second messenger that shapes a wide range of cellular functions including proliferation, migration, and cell differentiation in various cell types (Berridge et al., 2003). Increases of intracellular Ca^2+^ concentration are triggered by a variety of channels, transporters, and Ca^2+^-binding proteins (CaBP). These Ca^2+^ toolkit components encode specific Ca^2+^ signals that are defined by their spatio-temporal profile and their magnitude (Berridge, 1990; Berridge et al., 2003).

A growing bulk of evidence has pointed to a major role of Ca^2+^ in NSCs of the adult brain and in their pathological counterparts, namely GSCs. After a general overview of NSCs of the adult brain and on GSCs, we will review the progress that has been achieved on how Ca^2+^ signals regulate specific functions and properties of these cells. When appropriate, we put the data of the literature into the perspective of the development of treatments to combat brain diseases.

## Adult Brain Neural Stem Cells, Neurogenesis, and Pathology

### The Discovery of Neurogenesis and Neural Stem Cells in the Adult Brain

Most adult organs retain a population of somatic stem cells that ensure tissue homeostasis and repair by producing new cells in response to physiological conditions or injury. The brain has long been considered an exception to this rule: it was widely assumed that neurogenesis occurred only during embryogenesis and early postnatal life and that only glial cells could be produced in adulthood. This dogma was first challenged in the 1960s by Joseph Altman who reported addition of new neurons in the olfactory bulb and hippocampus of adult rats, but experimental evidence obtained with the tools available at that time was not robust enough to counter the dogma stating that neurogenesis does not occur in adults (Altman, 1963, 1969; Altman and Das, 1965). With the emergence of new tools to label proliferating cells, the persistence of neurogenesis throughout life was then directly demonstrated in the brain of adult songbirds (Nottebohm, 1985), rodents (Luskin, 1993; Lois and Alvarez-Buylla, 1994), and non-human primates. In these latter, incorporation of new neurons was described not only in the hippocampus (Gould et al., 1998, 1999; Kornack and Rakic, 1999) and olfactory bulb (Kornack and Rakic, 2001) but also amygdala (Bernier et al., 2002). Since then, neurogenesis has been identified in other brain areas like the hypothalamus of rodent and sheep brains (Kokoeva et al., 2005; Migaud et al., 2010; Yoo and Blackshaw, 2018) and the dorsal vagal complex in rodents (Bauer et al., 2005). In 1998, the analysis of brain tissue obtained post-mortem from patients, who had been injected with the nucleotide analog bromodeoxyuridine (BrdU) for diagnostic purposes during their cancer treatment, established that new neurons are generated throughout the lifespan from dividing progenitor cells in the dentate gyrus of adult humans (Eriksson et al., 1998). This discovery settled the relevance of adult neurogenesis for humans and attracted interest in the field. A neurogenic activity in the human hippocampus and olfactory bulb as well as in the striatum, a structure adjacent to V-SVZ, has thereafter been confirmed by several studies, although some controversy persists concerning the addition of new neurons in the adult human brain (Bédard and Parent, 2004; Sanai et al., 2004; Spalding et al., 2013; Ernst et al., 2014; Boldrini et al., 2018; Kempermann et al., 2018; Sorrells et al., 2018).

**Figure 1:**
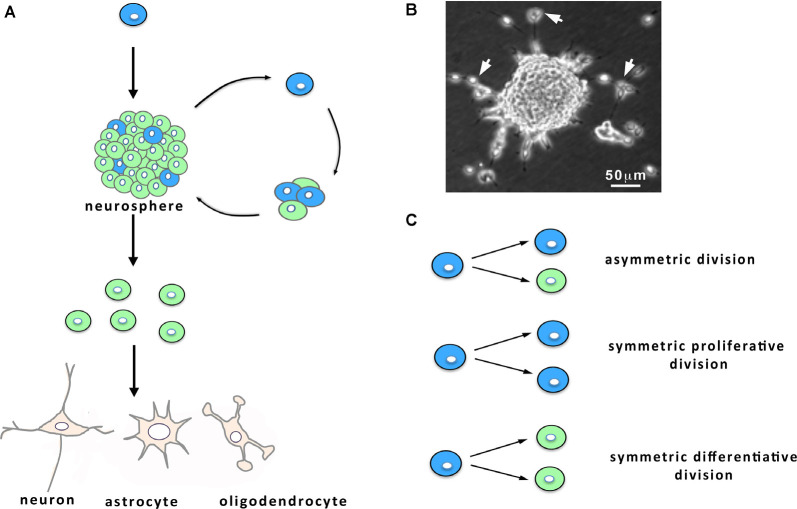
Characteristics of neural stem cell (NSC) cultures. **(A)** When maintained *in vitro* in the presence of growth factors, NSCs (in blue) from the ventricular-subventricular zone (V-SVZ) proliferate and give birth to both NSCs and progenitors (in green) that engender differentiated cells, namely neurons, astrocytes and oligodendrocytes. Within the cultures, the progenies derived from the NSC remain together and form neurospheres **(A,B)** from which can emerge following 1 week *in vitro* some migrating neuroblasts. **(B)** Micrograph of a culture showing a neurosphere and migrating neuroblasts (arrows). **(C)** NSC division: NSCs can undergo either asymmetric cell divisions, giving birth to a NSC (in blue) and a progenitor (in green), or symmetric proliferative cell divisions, engendering two NSCs, or symmetric differentiative cell divisions, leading to two progenitors.

In parallel to the discovery of neurogenesis in the brain of adult mammals, cells with NSC properties were isolated by Reynolds et al. (1992) from adult mice brain and propagated *in vitro*. When placed in appropriate cell culture conditions, NSCs proliferate, forming structures called neurospheres, self-renew, and subsequently give birth to neurons and glial cells (Figures 1A,B). Since this seminal work, NSCs have been obtained from a few adult rodent brain regions (including the hippocampus and the area postrema) of which the V-SVZ lining the brain lateral ventricles (LVs) contains the largest pool of dividing cells with defining characteristics of NSCs (Reynolds et al., 1992; Weiss et al., 1996; Palmer et al., 1997; Charrier et al., 2006). A decade later, Sanai et al. (2004) showed that V-SVZ harvested on post-mortem human brain contains multipotent NSCs that can be amplified *in vitro* and that retain the capacity to produce neurons and glial cells. Fueling considerable hope for stem cell-based brain therapies, these studies have been implemented by the demonstration of the possibility to harvest NSCs by endoscopy from human V-SVZ (Westerlund et al., 2005), which opens the opportunity to autologous cell transplantation and could help to circumvent the problems associated with the rejection of heterologous cell grafts.

### The V-SVZ Structure and Function

**Figure 2:**
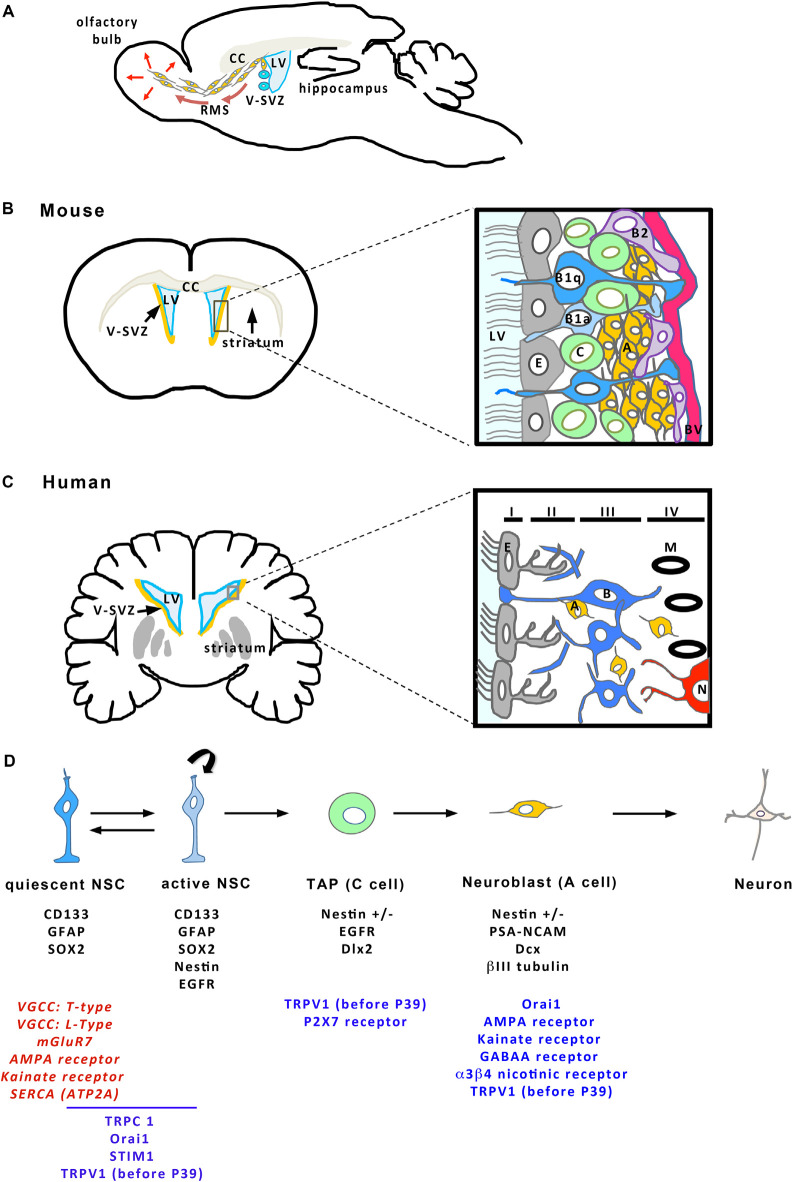
Cellular organization of the V-SVZ. **(A)** Sagittal view of the mouse brain showing the V-SVZ that borders the brain lateral ventricle (LV). The V-SVZ contains NSCs (in blue) producing neuroblasts (in orange) that migrate tangentially along the rostral migratory stream (RMS) to join the olfactory bulb where they integrate the existing network. CC, Corpus callosum. Frontal sections (on the left) of the mouse **(B)** and the human **(C)** brains represent the V-SVZ that borders the LVs. The square delineates the zone enlarged illustrating a schema of the organization of the V-SVZ (on the right). **(B)** (mouse brain) Ependymal cells (E, in gray) border the LV. NSCs (B1 cells in blue; B1a: active B1 cells; B1q: quiescent B1 cells) are located just beneath the ependymal layer and extend a cilium in the LV and a process that contacts a blood vessel (BV, in red). B1 cells can give birth to B2 cells (purple) that may act as NSC reserve cells. B1 cells engender transient amplifying progenitors (C cells, in green) that give birth to neuroblasts (A cells, in orange), then differentiating as neurons in the olfactory bulb. **(C)** In human brain, the V-SVZ is organized in four layers. Layer I is formed of ependymal cells (E, in gray). Layer II is a hypocellular layer with mainly processes. Layer III contains the cell bodies of astrocytes, part of which are NSCs (B cells, in blue). Layer IV is a transition layer with the parenchyma where myelin (M in black) and neurons (N, in red) can also be found. NSCs produce some neuroblasts (A cells, in orange) that are found across layers II to IV. **(D)** The different stages of adult neurogenesis from B cells to differentiated neurons. B cells (in blue) are NSCs that can shuttle between a quiescent and an active state. Neurogenesis occurs when B cells give birth to C cells that engender A cells migrating towards the olfactory bulb and then, differentiating as neurons. On the schema, characteristic markers expressed by the different cell types of the SVZ are indicated in black below each cell. The different components of the Ca^2+^ toolkit expressed within each of the cells are given below the corresponding cell. For B cells, some transcriptomic studies have highlighted the expression of specific components in quiescent cells. When the only information available concerns RNA expression, the corresponding actors are written in red, otherwise they are in blue. This figure has been elaborated according to the following references: Nguyen et al. (2003); Platel et al. (2008); Khodosevich et al. (2009); Young et al. (2010); Daynac et al. (2013); Sharma (2013); Khatri et al. (2014); Somasundaram et al. (2014); Stock et al. (2014); Domenichini et al. (2018); and Leeson et al. (2018).

The V-SVZ is a thin layer of cells extending along the walls of the LVs (Figures 2A,B). Combined immunocytochemical and ultrastructural characterization of the adult rodent V-SVZ enabled to recognize four main cell types within this germinal region: neuroblasts (Type A cells), SVZ astrocytes (Types B1 and B2 cells), immature precursors (Type C cells), and ependymal cells (Type E cells; Doetsch et al., 1997; Figure 2B). Ependymal cells form a single multiciliated layer that borders the LV and that contributes to the circulation of the cerebrospinal fluid flow. Type B cells possess ultrastructural and immunocytochemical staining characteristics of astrocytes, for example, GFAP (glial fibrillary acidic protein)-immunoreactivity, and express markers of immature cells such as SOX2 (Doetsch et al., 1997). Type B cells have been further subdivided into type B1 and type B2 cells based on their localization within the V-SVZ and cellular properties. Multiple lineage-tracing studies consistently identified type B1 cells as the NSC population (Doetsch et al., 1999a; Imura et al., 2003; Garcia et al., 2004). These cells display a radial morphology, have a short apical process with a single primary cilium sent into the LV, and extend a long basal process ending on blood vessels (Mirzadeh et al., 2008). The primary cilium, which is exquisitely poised to sense molecular signals and the mechanical flow of the cerebrospinal fluid, contributes to controlling NSC activation (Tong et al., 2014). In adult mice, there are roughly 6,000 B1 cells on the lateral wall of the LVs (Mirzadeh et al., 2008). NSCs can shuttle between activity (B1a) and quiescence (B1q), a process that is regulated by the signals arriving on NSCs. When activated, type B1 cells divide slowly, self-renew and give rise to type C cells that act as transient amplifying progenitors and express immature markers like nestin (Doetsch et al., 1999b; Figure 2D). For cell proliferation, NSCs can undergo three modes of division (Figure 1C): asymmetric divisions produce both a stem cell and a progenitor and thus couple NSC pool maintenance to neurogenic activity, symmetric proliferative divisions engender two NSCs, and symmetric differentiative divisions generate two progenitor cells. Analysis of NSC mode of division established that they mostly divide by symmetric divisions to either self-renew or produce differentiated progenies, indicating that neurogenesis and self-renewal might be independently regulated in the V-SVZ (Obernier et al., 2018). Type B2 cells have been poorly studied but a recent publication proposed that they may be produced by type B1 cells and serve as a reserve population of NSCs (Obernier et al., 2018). Type C cells divide rapidly and generate neuroblasts (type A cells; Figures 2B,D) that also undergo one or two rounds of cell division to further amplify the number of new neurons engendered (Ponti et al., 2013). Neuroblasts produced in the V-SVZ migrate for a long distance in aggregates forming tangentially oriented chains that coalesce into a defined pathway, the so-called rostral migratory stream (RMS; Figure 2A; Lois and Alvarez-Buylla, 1994; Lois et al., 1996). After about 5 days, neuroblasts reach the core of the olfactory bulb, detach from the chains and start to migrate radially towards their final location within the olfactory bulb (Petreanu and Alvarez-Buylla, 2002). Then, they differentiate as local interneurons that integrate the pre-existing neuronal network and contribute to olfactory processing and memory (Gheusi et al., 2000; Petreanu and Alvarez-Buylla, 2002; Tepavčević et al., 2011; Alonso et al., 2012; Gheusi and Lledo, 2014). In young adult mice, it has been estimated that NSCs produce approximately 10,000 young migrating neuroblasts every day (Doetsch et al., 1999b). NSCs also generate oligodendrocytes that join neighboring corpus callosum, striatum, and fimbria-fornix both in physiological conditions and after demyelination (Menn et al., 2006; Nait-Oumesmar et al., 2007; Ortega et al., 2013; Capilla-Gonzalez et al., 2014b). Although NSCs sustain lifelong neurogenesis and gliogenesis, it remains unknown whether individual NSCs are multipotent *in vivo* or whether neurons and glia arise from distinct NSCs (Menn et al., 2006; Capilla-Gonzalez et al., 2013; Ortega et al., 2013; Sohn et al., 2015).

The V-SVZ of adult humans is mainly similar to the V-SVZ of rodents described above but differs by its organization in four layers (Quiñones-Hinojosa et al., 2006; Figure 2C). Layer I is a monolayer of ependymal cells that border the ventricular lumen. This layer is followed by a hypocellular layer (Layer II), heavily populated with processes of astrocytes that are joined to each other by gap junctions and large desmosomes (Quiñones-Hinojosa et al., 2006). A dense ribbon of cell bodies, many of which belong to astrocytes form layer III, lies next to layer II. Some of these astrocytes proliferate and behave as multipotent stem cells (type B cells) when placed *in vitro* (Sanai et al., 2004; Quiñones-Hinojosa et al., 2006). Some isolated neuroblasts (type A cells) are also found within this layer as well as in layers II and IV, the latter being a transition layer with the brain parenchyma (Sanai et al., 2004; Macas et al., 2006; Curtis et al., 2007; Wang et al., 2011). The V-SVZ of children younger than 18 months of age harbors many neuroblasts migrating along specific pathways to integrate as neurons not only into the olfactory bulb but also into the ventral medial prefrontal cortex or the frontal lobe (Sanai et al., 2011; Paredes et al., 2016). This process has been reported to decline sharply during infancy (Sanai et al., 2011; Paredes et al., 2016). By adulthood, the existence of migrating neuroblasts along a RMS and incorporation of new neurons in the human olfactory bulb seems nearly extinct although there are conflicting results providing evidence of a maintenance neurogenic activity in the V-SVZ of the adult human brain (Curtis et al., 2007; Sanai et al., 2011; Wang et al., 2011; Paredes et al., 2016). One of the major hurdles when examining neurogenesis on post-mortem human brains is the possibility of labeling newly born cells. To circumvent this setback, the group led by Frisen measured ^14^C incorporated in neurons of post-mortem human brains to evaluate the numbers of newly incorporated cells in the adult brain. These studies are based on the fact that nuclear bomb testing during the Cold War in the 1950s produced elevated levels of ^14^C in the atmosphere, which entered the biotope and thereby, was incorporated in human molecules, including nucleotides forming DNA. It is assumed that the level of ^14^C in DNA mirrors the level of ^14^C at any given time, allowing the birthdate of cells in the human body to be determined retrospectively. This approach combined with histological studies showed that, in the adult human brain, V-SVZ neuroblasts robustly supply the adjacent striatum with new interneurons and also, generate new oligodendrocytes (Bergmann et al., 2012; Ernst et al., 2014). Altogether, these data support that the V-SVZ of the adult human brain contains NSCs whose implication in neurogenesis and gliogenesis *in vivo* requires further investigation in humans.

The NSCs and their activity are substantially affected by aging. Concretely, shrinkage of the pool of the V-SVZ NSCs occurs with age (Maslov et al., 2004; Shook et al., 2012; Kalamakis et al., 2019). This smaller stem cell reservoir seems to display a decreased proliferative activity (Bouab et al., 2011; Capilla-Gonzalez et al., 2014a; Kalamakis et al., 2019), although some reports indicated the opposite (Ahlenius et al., 2009; Shook et al., 2012). A recent study based on fluorescence-activated cell sorting (FACS) analysis and injury-induced regeneration experiments in mice nicely demonstrated that quiescent NSCs in the old brain are resistant to activation but, when activated, NSCs exhibit similar behavior in the brains of young and old mice (Kalamakis et al., 2019). This increase in quiescence during aging would allow protecting the stem cell reservoir from full depletion but makes old NSCs more resistant to regenerate the injured brain. Despite age-induced quiescence in NSCs, an increase in the number of progenitors has been reported in the aged adult human forebrain following ischemia, suggesting that NSCs, at least the active subpopulation of NSCs, might be recruited by injuries in the elderly (Macas et al., 2006). In addition to inducing a state of quiescence, aging also modifies the fate of the cells generated by NSCs, with a substantial reduction in the production of new neurons, whereas the genesis of oligodendroglial cells is unaffected in the aged brain (Ahlenius et al., 2009; Shook et al., 2012; Capilla-Gonzalez et al., 2013, 2015). Although the consequences of these alterations need further exploration, several reports suggest that impaired NSC activity may be associated with age-related diseases (Demars et al., 2010; Lazarov and Marr, 2013).

### Recruitment of Adult Brain Neural Stem Cells by Brain Pathologies

In addition to maintaining tissue homeostasis, somatic stem cells are defined by their ability to respond to lesions. In this regard, several studies disclosed that NSCs from the V-SVZ are recruited by various brain lesions including stroke and brain trauma, or by degenerative diseases (Gonzalez-Perez, 2012; Beckervordersandforth and Rolando, 2019; Bacigaluppi et al., 2020). To illustrate this concept, we chose to focus on two specific brain injuries: stroke, that is the second leading cause of death and the third leading cause of disability according to the World Health Organization, and multiple sclerosis, that is one of the most widespread disabling neurological condition of young adults around the world.

A stroke occurs when the blood flow to the brain is disrupted because of occlusion or rupture of a cerebral artery. Ischemia, caused by blockage of an artery, is the most common form of stroke. It leads to irreversible injury in the core region and partially reversible damage in the surrounding penumbra zone that has been deprived of blood supply. Stroke induction by transient middle cerebral artery occlusion, the most common model used for research in rodents, has been reported to boost cell proliferation in the V-SVZ by triggering NSC activation and to provoke the migration of neuroblasts from the V-SVZ towards the damaged areas (Arvidsson et al., 2002; Parent et al., 2002; Jin et al., 2003; Zhang et al., 2004). In the injured striatum, the neuroblasts arising from the V-SVZ differentiated as mature neurons, but 80% of the stroke-generated striatal neurons died during the first 2 weeks after their formation, which resulted in a very weak replacement of the striatal neuronal population lost due to the injury (Arvidsson et al., 2002). Although disappointing from a clinical perspective, these data point to the fact that NSCs contribute to attempts at brain repair. Furthermore, the fact that specific ablation of migrating neuroblasts in transgenic mice models resulted in increased infarct size and worsened functional recovery strongly suggests that stroke-recruited neuroblasts may help brain healing through mechanisms that are still elusive (Jin et al., 2010; Sun et al., 2012). It may be interesting to mention here that recent demonstrations of the ability of NSCs and neuroblasts to remove dead cell debris through phagocytosis raise a possible role of these cells in clearing the diseased environment, although this hypothesis needs to be experimentally challenged (Lu et al., 2011; Ginisty et al., 2015). Besides, NSC activation and an increase in the number of neuroblasts close to the lateral ventricular walls have also been described in brains of patients with ischemia, even those of advanced age (up to 80 years; Jin et al., 2006; Macas et al., 2006; Martí-Fàbregas et al., 2010). These results suggest that, in humans, the adult brain NSCs retain a capacity to respond to ischemic injuries and that this capacity is maintained in old age.

Multiple sclerosis is a chronic, autoimmune, and demyelinating disease that affects the central nervous system, especially among young individuals. In this pathology, most patients initially develop a clinical pattern with periodic relapses followed by remissions that ultimately end in permanent neurological disability. Experimental rodent models of demyelination displayed increased cell proliferation and migration of V-SVZ progenitors into the injured white matter where many of the mobilized cells differentiated as oligodendrocytes, in response to demyelination (Picard-Riera et al., 2002). Consecutive lineage tracing studies confirmed that V-SVZ cells, in addition to resident parenchymal oligodendroglial precursors, efficiently produce new oligodendrocytes with robust capacities of remyelination (Xing et al., 2014; Brousse et al., 2015). However, a recent study reported that selective ablation of V-SVZ cells although resulting in lowered oligodendrogenesis in mice models with multiple sclerosis, did not impact the ensuing remyelination but enhanced axonal loss (Butti et al., 2019), suggesting that NSCs may exert their beneficial effects either through oligodendrocyte replacement, or by providing trophic support, or both. In the human brain, NSC recruitment and oligodendrogenesis were also found in post-mortem brains of patients with multiple sclerosis and, importantly, were shown to occur in the brains of elderly patients (Nait-Oumesmar et al., 2007; Snethen et al., 2008). Interestingly, the olfactory dysfunction observed in mice models of multiple sclerosis and related to the reduced supply of olfactory bulb neurons was also described in patients, suggesting that at least part of the mechanisms identified in mice may occur in humans (Tepavčević et al., 2011).

### Adult Neural Stem Cell Relationship With Cancer Stem Cells in Brain Tumors

**Figure 3:**
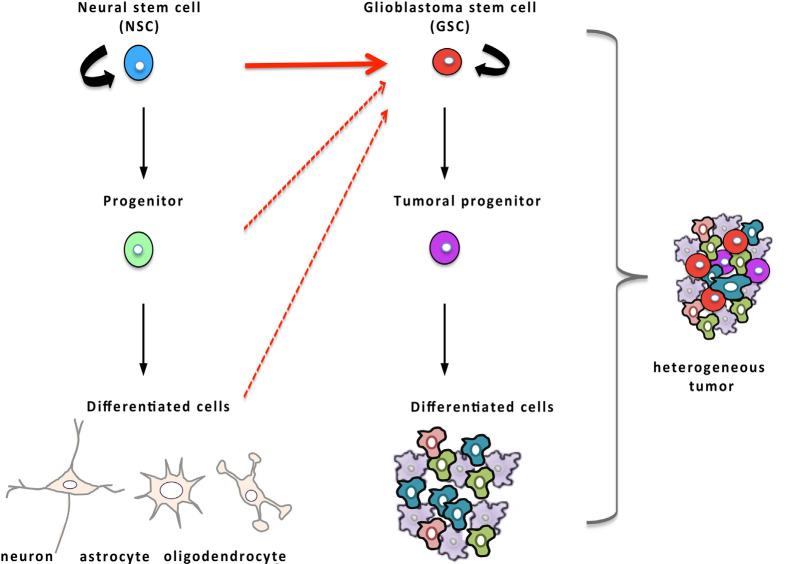
Glioblastoma stem cells (GSCs) and their relationship to NSCs. Hierarchical organization of tumors that, by analogy with NSCs (in blue) of the normal tissue displayed on the left part of the figure, possess, at the apex, GSCs (in red) endowed with the regenerative potential and capable of producing more differentiated tumor cells and recapitulating tumor heterogeneity. Compelling evidence established that oncogenic mutations in NSCs are responsible for the emergence of GSCs (red arrow) although some data suggest that GSCs may also arise from more differentiated cells (dashed red arrows).

Glioblastoma is the most frequent of primary brain tumors and among the most refractory of malignancies. Standard treatment includes gross total surgical resection followed by concurrent radiotherapy and chemotherapy with the drug temozolomide (Stupp et al., 2005). Because of the resistance of glioblastoma to the current treatments, the median survival of patients hardly reaches 15 months from the time of the diagnosis (Stupp et al., 2005). Thus, new means of eliminating these tumoral cells are keenly awaited. In the process of improving the understanding of glioblastoma growth and recurrence, a subpopulation of tumor cells, called GSCs, was identified as the cause of tumor initiation, resistance to current therapies, and thus, disease recurrence (Singh et al., 2004; Bao et al., 2006; Chen et al., 2012; Chesnelong et al., 2019). The cancer stem cell hypothesis states that tumors mimic normal tissues with hierarchically arranged populations of cells, with cancer stem cells at the apex that are endowed with regenerative potential and with the ability to provide all the functional diversity of the original tumor (Figure 3). As they are more quiescent than the other tumoral cells, cancer stem cells survive the current treatments that target highly proliferative cancer cells, and then, rebuild the tumor after the treatment, provoking cancer relapse in the patient.

Because GSCs share several properties with NSCs, the hypothesis that they may derive from NSCs has been explored (Figure 3). In the 1960s, it was reported that the offspring of pregnant rats, exposed to a single injection of the mutagenic molecule *N*-Ethyl-*N*-Nitrosourea during late gestation, developed spontaneous glioblastoma when they were 4-months-old. These animals displayed early neoplasia in the V-SVZ several weeks before macroscopic tumors were noted, suggesting that glioblastoma may originate in the V-SVZ germinal niche (Recht et al., 2003). The advent of genetic tools to specifically transfer oncogenes or to inhibit tumor-suppressors *in vivo* in NSCs of transgenic mice made it possible to assess and substantiate the idea that NSCs represent possible cells of origin of glioblastoma. To selectively infect NSCs, Holland and colleagues took advantage of the RCAS-TVA viral system to target nestin-positive stem cells *in vivo*. In this approach, the gene coding for avian retrovirus receptor TVA is placed under the control of the nestin promoter which leads to an expression of TVA selectively in nestin-positive cells in the transgenic mice, allowing thereafter transduction of dividing nestin-positive cells with the avian retrovirus RCAS through its receptor TVA. The introduction of an RCAS viral vector containing the oncogenic forms of both Ras and Akt in these transgenic neonatal mice resulted in the spontaneous onset of high-grade glioma (Holland et al., 2000). About a decade later, Marumoto et al. (2009) confirmed that targeting oncogenic mutations in NSCs of the V-SVZ leads to the development of glioblastoma. To this end, they took advantage of the cre/lox system to drive the expression of oncogenic Ras or Akt specifically in GFAP-expressing cells of the V-SVZ. For that purpose, they injected lentiviral vectors encoding of oncogenic Ras or Akt with expression controlled by cre recombinase in the V-SVZ of adult immunocompetent mice, heterozygous for p53 and genetically engineered to have a GFAP-driven expression of the recombinase cre. In these mice, the cre-induced expression of oncogenic Ras or Akt in GFAP-positive cells of the V-SVZ provoked the development of high-grade glioma (Marumoto et al., 2009). This study moreover underlined that infection of as few as 60 GFAP-expressing cells in the V-SVZ was sufficient to induce the emergence of a full-blown tumor containing GSCs whereas glioblastoma rarely developed when GFAP-expressing non-stem cells from the cortex were transduced (Marumoto et al., 2009). Concomitant studies corroborated that introduction of glioblastoma-specific oncogenic mutations in NSCs of the V-SVZ lead to a spontaneous tumoral development with a 100% penetrance (Alcantara Llaguno et al., 2009; Jacques et al., 2010). Altogether, these data consistently support that high-grade glioma originates from NSCs of the V-SVZ that have undergone malignant transformation although a few reports have identified more differentiated cells as an alternative source of cells at the origin of glioblastoma (Lindberg et al., 2009; Liu et al., 2011; Friedmann-Morvinski et al., 2012).

Recently, a study based on deep-sequencing analysis of brain tissues provided evidence that oncogenic mutations in the V-SVZ are responsible for the development of high-grade glioma in humans (Lee et al., 2018). The publication reports that, in more than half of the human brains examined, normal SVZ tissue away from the tumor contained low-level cancer-deriving mutations in genes such as TP53, PTEN, EGFR, and TERT promoter, that were similar to those found in their matching tumors (Lee et al., 2018; Matarredona and Pastor, 2019). Furthermore, the authors described that NSCs from the V-SVZ carrying driver mutations were able to migrate from the SVZ and to form high-grade malignant gliomas in distant brain regions, clearly suggesting an ontogenetic link between NSCs from the V-SVZ and glioblastoma that has been confirmed by other studies (Garcia and Dhermain, 2018; Lee et al., 2018; Tejada Neyra et al., 2018). Accordingly, it is currently admitted that cell-autonomous changes in NSCs of the V-SVZ germinal zone are most likely responsible for the emergence of glioblastoma and that NSCs could represent the cells of origin of GSCs, which matches actual demographic data on glioma incidence in the human population (Bauer et al., 2014).

In addition to this ontogenetical relationship between NSCs and GSCs, direct interactions between NSCs and the tumor have been found. It was discovered in experimental mice models of glioma that NSCs are endowed with a tumor tropism that allows them to track tumor cells and circumscribe the tumor mass, overall leading to an oncostatic effect and improved survival of mice bearing the tumor (Glass et al., 2005). This innate tumor tropism of NSCs for glioblastoma is currently exploited in clinical trials using genetically engineered therapeutic NSCs to deliver cytotoxic drugs specifically to the tumor site (Portnow et al., 2017; Gutova et al., 2019).

## The Calcium Toolkit

**Figure 4:**
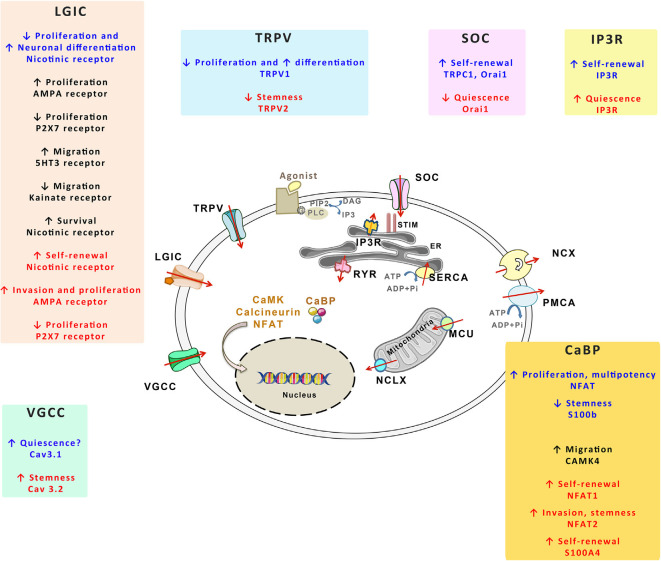
The Ca^2+^ toolkit in NSCs and GSCs. The figure summarizes the different components of the Ca^2+^ toolkit. The red arrows represent the Ca^2+^ flux. Plasma membrane channels include voltage-gated Ca^2+^ channels (VGCCs), ligand-gated ion channels (LGICs), transient receptor potential vanilloids (TRPVs) and store-operated channels (SOCs). Plasma membrane also contains Ca^2+^-extruding mechanisms, namely Na^+^/Ca^2+^ exchanger (NCX) and plasma membrane Ca^2+^ ATPase (PMCA). Within the endoplasmic reticulum (ER), both IP3 receptor (IP3R) and ryanodine receptor (RYR) allow Ca^2+^ release in the cytosol whereas sarco/endoplasmic reticulum Ca^2+^-ATPase (SERCA) replenishes ER Ca^2+^ stores. STIM1 detects ER Ca^2+^ concentrations that activate SOC. Mitochondria release or take up Ca^2+^ respectively through mitochondrial Na+/Ca^2+^ exchanger (NCLX) and mitochondrial Ca^2+^ uniporter (MCU). The cells also contain Ca^2+^ binding protein (CaBP) that act as Ca^2+^ buffers or that transduce Ca^2+^ signals (calcineurin, NFAT, CaMK). GPCR corresponds to G protein coupled receptors, PLC to phospholipase C. For each of these Ca^2+^ actors, their effects on NSCs (written in blue), on the progenies of NSCs (written in black) and on GSCs (written in red) are provided within the specific insets.

NSCs and GSCs, like the other cells of the organism, evolve in specific microenvironments whose signals shape their activities in response to the physiological demand or the pathological growth. Numerous microenvironmental signals transduce their effects through transitory rises of intracellular concentration of Ca^2+^ with specific durations and magnitudes, either in discrete cellular microdomains or throughout the whole cell (Berridge et al., 2003). Because of its wide repertoire of spatio-temporal variations in its intracellular concentrations, the Ca^2+^ signal is ideally poised to determine cellular processes in response to extracellular signals. Consequently, strict handling of Ca^2+^ is required to maintain optimized cellular functions. Ca^2+^ fluctuations are managed by different molecules that can be subdivided as follows: Ca^2+^ permeable channels that flux Ca^2+^ into the cell through the plasma membrane or that release Ca^2+^ from internal stores of the endoplasmic reticulum (ER) and mitochondria, Ca^2+^ pumps and exchangers that replenish Ca^2+^ stores or that extrude Ca^2+^ from the cell, and CaBP that buffer cytosolic free Ca^2+^ or act as effectors. The coordinated action of the Ca^2+^ toolkit components maintains low resting levels of cytosolic free Ca^2+^ and encodes specific Ca^2+^ signals that trigger selective cellular activities in response to extracellular signals (Berridge et al., 2003). While the Ca^2+^ signal is ubiquitously used to transduce extracellular signals, the proteins that control Ca^2+^ transport display cell type-specific expression, allowing selective cellular responses. Compelling evidence suggests that Ca^2+^ channels play a pivotal role in tumor biology, display aberrant expression or localization and that they are hijacked to promote tumor growth and resistance to treatment (Déliot and Constantin, 2015; Prevarskaya et al., 2018; Terrié et al., 2019). Figure 4 shows an overview of the key components of the Ca^2+^ toolkit. Here below, we provide a short description of this toolkit.

### Plasma Membrane Channels

At rest, the cytosolic concentration of Ca^2+^ is maintained at very low levels (50–100 nmol/L), which is about 10,000-fold less than those of the extracellular medium. Thus, the opening of any Ca^2+^ channel located in the plasma membrane leads to a pronounced Ca^2+^ influx that sharply raises the intracellular Ca^2+^ concentration, that in turn, affects the activity of nearby proteins and elicits biological responses (Samanta and Parekh, 2017). Ca^2+^ inflow from the external milieu is controlled by a variety of transmembrane proteins, of which Ca^2+^ channels mediate Ca^2+^ entry from an extracellular compartment in response to diverse stimuli: membrane depolarization, extracellular agonists, intracellular messengers, mechanical stretch, or depletion of intracellular Ca^2+^ stores (Berridge et al., 2003).

#### Voltage-Gated Calcium Channels (VGCCs)

VGCCS also called voltage-operated calcium channels (VOCC) mediate Ca^2+^ influx in cells in response to membrane depolarization. An initial increase of the membrane potential is required for the activation of the intrinsic voltage sensor and subsequent pore opening. VGCCs comprises ten members, based on the expression of a specific pore-forming α_1_-subunit containing the voltage sensor of the Cav family channels. This family has been subdivided into three phylogenetic subfamilies according to the characteristics of the currents they mediate: Ca_V_1 subfamily channels that give rise to L-Type currents, Ca_V_2 subfamily channels that produce to P/Q-, N-, and R-type currents, and Ca_V_3 subfamily channels that mediate T-type currents (Catterall et al., 2005; Catterall, 2011). L-type and T-type Ca_V_ subfamilies are expressed in many cell types while N, P/Q, and R-type channels are predominantly expressed in neurons. VGCCs have been first studied in electrically excitable cells, but it is now clear that they are functionally expressed in non-excitable cells including various cancer cell types (Prevarskaya et al., 2010, 2018).

### Receptor-Operated Channels (ROCs) and Store-Operated Channels (SOCs)

#### ROCs

ROCs are switched on *via* ligand-mediated activation of receptors coupled to the phospholipase C (PLC) that breaks down PIP2 to produce diacylglycerol (DAG) and inositol 1,4,5-trisphosphate (IP3). DAG activates plasma membrane channels from the TRPC (Transient receptor potential canonical) subfamily, leading to a cationic current (Na^+^ and Ca^2+^), a process referred to as receptor-operated Ca^2+^ entry (ROCE; Vazquez et al., 2001; Dietrich et al., 2005). Because DAG is a second messenger, this channel type can be also called Secondary Messenger-Operated Channels (SMOCs). Among the seven TRPC identified, homotetrameric TRPC3, TRPC6 or TRPC7 channels and heterotetrameric TRPC1–TRPC3 and TRPC3–TRPC4 channels can open in response to DAG application, resulting in Ca^2+^ influx (Hofmann et al., 1999; Lintschinger et al., 2000; Liu et al., 2005a; Poteser et al., 2006). In contrast, homomeric TRPC1, -4, and -5, which are also activated by receptor-induced PLC, are completely unresponsive to DAG (Hofmann et al., 1999; Venkatachalam et al., 2003).

#### SOCs

Also known as capacitive Ca^2+^ entry, store-operated Ca^2+^ entry (SOCE) represents a widespread type of Ca^2+^ entry that is triggered upon IP3-dependent depletion of ER Ca^2+^ stores (Parekh and Putney, 2005). The ER transmembrane protein Stromal Interaction Molecule 1 (STIM1) senses Ca^2+^ decrease in the ER and opens SOCs to replenish intracellular Ca^2+^ stores through SERCA (Sarco/endoplasmic reticulum Ca^2+^-ATPase) and elicit cellular responses (Parekh and Putney, 2005). Initially identified as CRAC (Ca^2+^ Release-Activated Channels) in T cells, Orai1 homopolymers form the core component of SOCs. Although Orai1 is the ubiquitous SOC protein, Orai2 and Orai3 mediate SOCE in certain specific tissues (Motiani et al., 2010; Wang et al., 2017c). Depending on the cell type and on the STIM1/TRPC channel ratio (Lee et al., 2010), SOCE and I_SOCE_ (the current produced by SOCE) can also be supported by TRPC tetrameric channels (Lopez et al., 2020), which can be formed of TRPC1 and TRPC4 (Sabourin et al., 2009, 2012; Antigny et al., 2017), that interact directly with STIM1 (Huang et al., 2006; Yuan et al., 2007). SOCE and cationic I_SOCE_ can also be the result of functional cooperation between TRPC1 with Orai1 (Huang et al., 2006; Jardin et al., 2008; Desai et al., 2015; Shin et al., 2016; Ambudkar et al., 2017). SOCE has been recognized as an essential mechanism for Ca^2+^ uptake in non-excitable cells that, in addition to restoring ER Ca^2+^ stores, elicits Ca^2+^-dependent intracellular signaling cascades involved in cell proliferation, migration, and differentiation.

Further, Orai3 can form a heteromultimeric channel with Orai1 to build-up arachidonic acid-regulated Ca^2+^ channels (ARC) or arachidonic acid metabolite Leukotriene C4 (LTC4)-regulated Ca^2+^ channels (LRC; Shuttleworth, 2009; González-Cobos et al., 2013). Different works suggest that Orai3 regulates breast, prostate, lung, and gastrointestinal cancers by either forming SOC or ARC/LRC channels in the cancerous cells but not in healthy tissue (Tanwar et al., 2020). Additionally, a recent study reinforces the idea that native CRAC channel is formed by heteromultimerization of Orai1 with Orai2 and/or Orai3, in which Orai2 and Orai3 may act as Orai1 negative regulators (Yoast et al., 2020). Their role would be therefore critical to adapt Ca^2+^ signals to agonist strengths as proposed for Orai2 in T cells (Vaeth et al., 2017).

#### Ligand-Gated Ion Channels (LGICs)

LGICs also commonly referred to as ionotropic receptors, are ion channels whose gating is ligand-dependent. Neurotransmitters serve as ligands for ionotropic receptors. LGICs are opened, or gated, by the binding of a neurotransmitter that triggers a conformational change and results in the conducting state. The LGICs comprise the excitatory, cation-selective, nicotinic acetylcholine- (nAchR; Millar and Gotti, 2009), 5-HT_3_ serotonin- (Barnes et al., 2009), ionotropic glutamate- (Lodge, 2009), the inhibitory, anion-selective, GABA_A_- (Olsen and Sieghart, 2008) and glycine receptors (Lynch, 2009) as well as P2X receptors (Jarvis and Khakh, 2009) gated by extracellular adenosine 5’-triphosphate (ATP; North, 2016). In the central nervous system, activation of P2X receptors supports Ca^2+^ entry into neurons and modulates neuron responses. It is also well known that extracellular ATP is abundant in the tumor microenvironment, and since ATP may be leaked by damaged cells, stimulation of P2X channels may be prevalent upon traumatic or ischemic injury (Puchałowicz et al., 2014).

#### TRPV (Transient Receptor Potential Vanilloid) Channels

TRPVs are Ca^2+^ permeable channels of the TRP family (Pedersen and Nilius, 2007) that respond to various signals from the microenvironment such as exogenous chemical ligands, noxious heat, or mechanical stretch. Because of these properties, various channels of the TRPV family, that contains six mammalian members, are known to be involved in nociception (Patapoutian et al., 2009), taste perception (Nilius and Appendino, 2013), thermosensation (Tominaga, 2007) or mechano and osmolarity sensing (Guilak et al., 2010). While heat-activated TRPV1-TRPV4 channels that also function as chemosensors are nonselective for cations and modestly permeable to Ca^2+^, TRPV5 and TRPV6 are highly Ca^2+^ selective channels with low-temperature sensitivity. Among TRPV channels, TRPV2 is a growth factor-regulated cation channel that was shown to be up-regulated in some cancer cell types, suggesting an oncogenic role, while down-regulated in other tumor cells, suggesting an opposite tumor suppression role (Liberati et al., 2014).

### Intracellular Ca^2+^ Handling and Ca^2+^ Signaling Pathways

Ca^2+^ handling by intracellular reservoirs and transporters may also impact cellular activity. The major Ca^2+^ store is the ER that releases Ca^2+^ to the cytoplasm, through two related Ca^2+^ release channels, namely the ryanodine receptors (RYRs) and inositol 1,4,5-trisphosphate receptors (IP3Rs) in response to multiple factors that include free Ca^2+^ ion. Three different isoforms of RYRs (RYR1, RYR2, RYR3), each encoded by a different gene, have been identified (Santulli et al., 2018). More widely expressed in most non-excitable cells than RYRs, IP3Rs, displaying three IP3R subtypes, are activated by IP3 produced in response to receptors in the plasma membrane that stimulate phospholipase C (PLC; Mikoshiba, 2015; Berridge, 2016). Specifically, the cell-surface receptors responsible for IP3 formation belong to two main classes, the G protein-coupled receptors (GPCRs) protein and the receptor tyrosine kinases (RTKs) that are coupled to different PLC isoforms (the PLCβ and PLC-γ isoforms respectively). During the transduction process, the precursor lipid PIP2 is hydrolyzed by PLC to produce both IP3 and DAG. The IP3 released from the membrane diffuses into the cytosol where it binds the IP3Rs, which open and releases Ca^2+^ from the ER. The subsequent Ca^2+^ release through IP3Rs as well as the propagation of the Ca^2+^ signal occurs in a wide range of cells. The Ca^2+^-mobilizing function of IP3 is terminated through the metabolization of IP3.

Ca^2+^ entry and/or Ca^2+^ release through the various channels leads to an increase of cytosolic free Ca^2+^ concentration whose spatio-temporal pattern and magnitude differentially activate specific signaling pathways (Samanta and Parekh, 2017). These latter rely on various CaBP of the cytosol, such as calmodulin, calcineurin, PKC, S100 proteins, parvalbumin (Yáñez et al., 2012). While some of them, like parvalbumin or S100, buffer free Ca^2+^, most of CaBP decode the Ca^2+^ signal by selectively turning on specific intracellular signaling cascades (Schwaller, 2010; Yáñez et al., 2012). For instance, the Ca^2+^ sensor calmodulin modifies its interaction with many effectors upon binding to Ca^2+^, that in turn, trigger distinct signaling pathways such as mitogen-activated protein kinase/extracellular signal-regulated kinase (MEK/ERK), Nuclear factor of activated T-cells (NFAT) or Ca^2+^/calmodulin-dependent protein kinase (CAMK) pathways (Hogan et al., 2003). A characteristic feature of the IP3 signaling pathway is that it usually generates a brief transient Ca^2+^ release that can be repeated to give oscillations over a longer period (Berridge, 1990; Fewtrell, 1993). Many functions, such as gland secretion or differential gene transcription, are controlled by frequency coding of Ca^2+^ oscillations that, in turn, differentially regulate CaBPs. For instance, the transcription factor nuclear factor kappa B (NFκB) was shown to be preferentially activated by low frequency repeated Ca^2+^ transients, whereas NFAT activation needs higher frequencies and a long duration Ca^2+^ signals (Dolmetsch et al., 1998). The activity of the CAMK II was also reported to be dependent on the frequency of Ca^2+^ oscillations (De Koninck and Schulman, 1998).

## Physiological Roles of Ca^2+^ in NSCs and Their Progenies

NSCs are subjected to a wide range of extracellular cues, each of which contributes to regulate NSC activity. Analysis of the transcriptome of purified adult NSCs highlighted the importance of Ca^2+^-dependent signaling pathways in these cells (Beckervordersandforth et al., 2010). In parallel, Ca^2+^ imaging experiments unveiled the existence in NSCs, of intercellular Ca^2+^ waves that are propagated through gap junctions and that are used by NSCs to communicate with each other and with neighboring niche astrocytes both under physiological conditions (Lacar et al., 2011) and pathological conditions (Kraft et al., 2017). Live cell Ca^2+^ imaging has also allowed to observe spontaneous Ca^2+^ oscillations in NSC/progenitors from the V-SVZ (Domenichini et al., 2018) as well as spontaneous high-frequency transients in migrating neuroblasts (Maslyukov et al., 2018). Along with the prominence of transcripts related to the Ca^2+^ pathway in NSCs, these data lend support to the essential role of Ca^2+^ in NSCs and their progeny. In the subsequent sections, we will review and discuss the involvement of the Ca^2+^ toolkit in NSC physiology by analyzing first the implication of plasma membrane channels and then, the function of intracellular Ca^2+^ handling and signaling pathways. An overview of the Ca^2+^ proteins expressed in NSCs and their functions are summarized in Table 1 and in Figures 2D, 4.

**Table 1:** Effects of the Ca^2+^ toolkit components on neural stem cells (NSCs) and Glioblastoma stem cells (GSCs).

	Neural stem cells	Glioblastoma stem cells
VGCC	Maintain quiescence (T-type, L-type) induced by GABA? ^(1, 2)^	Increase stemness and survival (T-type) ^(18, 19)^
SOC	Increase proliferation and self-renewal (TRPC1, Orai1) ^(3, 4)^	Increase proliferation ^(20)^
TRPV	Decrease proliferation and increase neuronal differentiation (TRPV1) ^(5)^	Increase cell death (TRPV1) ^(21)^
		Decrease stemness (TRPV2) ^(22)^
LGIC	Nicotinic receptors: decrease proliferation and increase neuronal differentiation ^(6, 7)^	Nicotinic receptors: increase stemness and clonogenicity ^(23)^
	NMDA receptors : increase oligodendroglial differentiation, increase neuroblast survival ^(8, 9)^	
	AMPA receptors : increase neuroblast proliferation ^(10)^	AMPA receptors: increase proliferation and invasion ^(24, 25)^
	Kainate receptors : decrease neuroblast migration ^(11)^	
	5HT3: increase neuroblast migration ^(12)^
	P2X7 receptors: decrease transient amplifying progenitor (C cell)
	P2X7 receptors: decrease proliferation ^(26)^
	proliferation ^(13)^
		
IP3R	Increase self-renewal ^(14)^ Increase cell death ^(15)^	Increase quiescence ^(27)^
CaBP	NFAT: Increase proliferation ^(16)^ S100B : decrease stemness ^(17)^	NFAT1 : increase self-renewal and stemness ^(28)^ NFAT2 : increase clonogenicity ^(29)^ S100A4 : increase self-renewal ^(30)^

*The table summarizes the major effects reported for Ca^2+^ toolkit actors in NSCs and their progenies and in GSCs according to the following references: 1. Young et al. (2010); 2. Daynac et al. (2013); 3. Somasundaram et al. (2014); 4. Domenichini et al. (2018); 5. Stock et al. (2014); 6. Narla et al. (2013); 7. Wang et al. (2017b); 8. Cavaliere et al. (2012); 9. Platel et al. (2010); 10. Song et al. (2017); 11. Platel et al. (2008); 12. García-González et al. (2017); 13. Leeson et al. (2018); 14. Kraft et al. (2017); 15. Shi et al. (2005); 16. Serrano-Pérez et al. (2015); 17. Raponi et al. (2007); 18. Niklasson et al. (2017); 19. Zhang et al. (2017); 20. Aulestia et al. (2018), 21. Stock et al. (2014); 22. Morelli et al. (2012); 23. Spina et al. (2016); 24. Venkataramani et al. (2019); 25. Venkatesh et al. (2019); 26. D’Alimonte et al. (2015); 27. Zeniou et al. (2015); 28. Jiang et al. (2019); 29. Song et al. (2020); 30. Chow et al. (2017)*.

### Plasma Membrane Channels

#### Voltage-Gated Calcium Channels (VGCCs)

Both the L-type Ca_V_1.2 and the T-type Ca_V_3.1 channels have been detected by RT-PCR in V-SVZ cell cultures that are formed of NSCs and their progenies (Kong et al., 2008). Subsequent analysis of prospectively purified NSCs by flow cytometry disclosed that quiescent NSCs display high expression levels of ion channels among which T- and L-type voltage-gated channels (Khatri et al., 2014). Indeed, electrophysiological recordings combined with pharmacology identified in NSCs (B cells), L- and T-type currents that could be physiologically evoked by GABA (Young et al., 2010). Constitutively present in the germinal niche, GABA that is produced by neuroblasts, tonically activates GABA_A_ receptors expressed by NSCs to maintain a quiescent state (Liu et al., 2005b; Daynac et al., 2013; Khatri et al., 2014). GABA_A_ receptors are ionotropic receptors that upon interaction with their neurotransmitter activate a depolarizing chloride current in NSCs whose resting membrane potential is close to −80 mV. It was shown that the depolarization elicited by GABA opens VGCCs, which provokes a Ca^2+^ flux, and that this Ca^2+^ inflow can be suppressed by the L-Type channel blocker nifedipine or the T-type inhibitor mibefradil (Young et al., 2010). Thus, Ca_V_ channels whose expression is enriched in quiescent NSCs (Khatri et al., 2014) could be key players in preventing NSC activation and in preserving their quiescent state in response to the ambient GABA of the V-SVZ. Although this hypothesis requires formal demonstration, the above data suggest that Ca_V_, by acting on NSC quiescence, would prevent excessive activation of NSCs and avoid NSC exhaustion.

In addition to NSCs, neuroblasts within the olfactory bulb also have been found to possess L-type channels that induce Ca^2+^ transients whose functions are yet undefined (Darcy and Isaacson, 2009).

#### Store-Operated Channels (SOCs)

In the V-SVZ, both Orai1 and TRPC1 have been detected along with STIM1 in NSCs and also in some neuroblasts (Skibinska-Kijek et al., 2009; Somasundaram et al., 2014; Domenichini et al., 2018). In these cells, SOCE could be triggered by various extracellular signals such as EGF that are used for cultures of NSCs (Reynolds et al., 1992; Somasundaram et al., 2014) or SDF1 that controls the tumor tropism of NSCs to glioblastoma (Zhao et al., 2008; Somasundaram et al., 2014). The cholinergic agonist muscarine that stimulates NSC self-renewal could also elicit an intracellular Ca^2+^ spike followed by a plateau phase that was dependent on SOCE (Domenichini et al., 2018). Conversely, SOCE was significantly but not totally reduced following the Orai1 knock-out (Somasundaram et al., 2014), indicating that Orai1 and other actors, possibly TRPC1, build-up functional SOCs in mouse NSCs. The pharmacological blockade of SOC or genetic deletion of Orai1 resulted in decreased cell proliferation in the V-SVZ without affecting cell death (Somasundaram et al., 2014; Domenichini et al., 2018). Importantly, recruitment of SOC by glutamate or muscarine (Giorgi-Gerevini et al., 2005; Domenichini et al., 2018) promoted NSC self-renewal while SOCE inhibition shifted the mode of NSC division from symmetric proliferative to asymmetric, suggesting that SOCs are required to maintain or expand the pool of NSCs (Domenichini et al., 2018). How SOCs control the mode of cell division is yet unknown and could rely on the ability of SOCE to mobilize specific Ca^2+^ actors or depend on the capacity of STIM and Orai to localize at the cleavage furrow (Chan et al., 2016).

The importance of SOCs in NSCs is further underscored by a recent study showing that SOCE is required for the transduction of mechanical information due to cerebrospinal fluid flow (Petrik et al., 2018). Specifically, the authors of that study reported that the epithelium sodium channel (ENaC) that is located in the primary cilium of NSCs acts as a mechanosensor and requires a subsequent SOCE to control cell proliferation in V-SVZ in response to cerebrospinal fluid flow (Petrik et al., 2018). Altogether, these data highlight that SOCs play a pivotal role in linking NSC activity to extracellular cues. Of interest, it has been shown in neurons, that after store depletion, STIM1 suppresses the activity of Ca_V_ 1.2 and Ca_V_ 3.1 VGCCs, both of which are expressed in quiescent NSCs and may contribute to maintaining a quiescent state (Harraz and Altier, 2014). Furthermore, it has been shown in muscle cells that SOC and Ca_V_ can be found in complexes, which would facilitate the switch for recruitment of either SOC or Ca_V_ (Ávila-Medina et al., 2016). If STIM1 holds a similar function in NSCs, this observation raises the assumption that STIM1 may orchestrate the shuttling between activity and quiescence of NSCs, by acting both on SOCs to trigger self-renewal and Ca_V_ channels to release NSCs from the quiescent state constitutively induced by ambient GABA.

#### Ligand-Gated Ion Channels (LGICs)

Because NSC activities are tightly controlled by neurotransmitters, several Ca^2+^-coupled LGICs have been identified as NSC regulators. For instance, NSCs possess, in addition to cholinergic muscarinic receptors that yield transient Ca^2+^ signals partly through SOCs (Somasundaram et al., 2014; Domenichini et al., 2018), ionotropic nAChRs of the α3 and α4 subtype (Paez-Gonzalez et al., 2014). Also, several studies indicate that other nAChR, namely α7 receptors are also expressed in V-SVZ and that neuroblasts, as well as possibly transient amplifying progenitors, display nAChR (Narla et al., 2013; Sharma, 2013). These receptors are physiologically exposed to acetylcholine in the V-SVZ niche that comes from innervation by cholinergic fibers from the basal forebrain (Calzà et al., 2003; Cooper-Kuhn et al., 2004) and that is also released by a small population of cholinergic neurons residing within the rodent V-SVZ (Paez-Gonzalez et al., 2014). Activation of the local cholinergic neurons or acute infusion of nicotine significantly promoted neurogenesis *in vivo* (Mudò et al., 2007; Paez-Gonzalez et al., 2014). Conversely, the destruction of basal forebrain cholinergic neurons impeded the production of new neurons (Calzà et al., 2003; Cooper-Kuhn et al., 2004), indicating that ligand-gated cholinergic receptors favor neurogenesis. The use of selective pharmacological and genetic tools identified that α7 nicotinic receptors while promoting neuronal differentiation, inhibit proliferation of V-SVZ cells under both physiological conditions or in response to ischemia whereas the β2-nAChR subunit control cell survival of newborn neurons (Narla et al., 2013; Wang et al., 2017a, b). Collectively, these studies lend support to a regulatory role of nicotinic receptors in the neurogenic activity of the healthy and diseased brain, albeit the pharmacology of these effects would merit further investigation.

NSCs and neuroblasts also express ionotropic glutamate receptors of the N-methyl-D-aspartate (NMDA), a-amino-3-hydroxy-5-méthylisoazol-4-propionate (AMPA), and kainate subtypes (Platel et al., 2008, 2010; Khatri et al., 2014). Although little is known about the functional roles of glutamate receptors in NSCs, kainate and AMPA receptors that trigger Ca^2+^ influx have been found in neuroblasts where they, respectively, reduce the speed of the migration along the LV (Platel et al., 2008) or boost proliferation as well as promote the ability of these cells to improve brain repair following stroke (Song et al., 2017). In contrast, NMDA receptors seem critical for neuroblast survival during their journey in the RMS before entering the olfactory bulb synaptic network (Platel et al., 2010). Physiologically, the glutamate supply that activates the migrating neuroblasts is secreted by astrocytes, suggesting that astrocytes use glutamate signaling to control the number of adult-born neurons reaching their final destination (Platel et al., 2010). NMDA receptors have also been shown to stimulate oligodendrocyte differentiation in V-SVZ stem/progenitor cells (Cavaliere et al., 2012). Furthermore, AMPA receptors have been detected in oligodendroglial progenitors derived from the V-SVZ during the regeneration process occurring after the demyelination of the corpus callosum (Etxeberria et al., 2010). Collectively, these data indicate that glutamate, through the activation of specific subsets of ligand-gated receptors, controls multiple steps of neurogenesis and gliogenesis in the adult brain.

The V-SVZ also harbors serotoninergic receptors that support large rhythmic Ca^2+^ oscillations in neuroblasts (García-González et al., 2017). Knock-out of the 5HT3A receptor severely reduced Ca^2+^ spikes, indicating that 5HT3A receptors represent a major gate for Ca^2+^ entry in migrating neuroblasts. Indeed, the speed of migration of neuroblasts was enhanced following optogenetic activation of serotoninergic fibers while it was disrupted by loss-of-function mutation of 5HT3A receptors (García-González et al., 2017), implying that serotoninergic innervation arising from the raphe nuclei governs neuroblast migration through Ca^2+^ inflow mediated by ionotropic 5HT3A receptors (García-González et al., 2017).

In addition to neurotransmitters, V-SVZ cells mobilize Ca^2+^ in response to extracellular nucleotides whose concentrations increase following brain injury because of their leakage by damaged cells. In addition to their roles in pathological states, nucleotides may also control constitutively the neurogenic niche where high levels of NTDPase2, the enzyme that hydrolyzes nucleotides, have been detected (Braun et al., 2003). Knock-out of NTDPase2 resulted in increased numbers of intermediate progenitors in the V-SVZ, suggesting that increased levels of extracellular nucleotides favor proliferation in the V-SVZ (Braun et al., 2003). Extracellular nucleotides mediate their effects through metabotropic G-protein-coupled P2Y purinergic receptors and ionotropic purinergic P2X receptors, both of which are expressed in the V-SVZ and induce Ca^2+^ transients (Stafford et al., 2007; Messemer et al., 2013). Specifically, the P2X7R subtype that has been detected in ependymal (E) is also expressed in transient amplifying progenitors (C cells) where it plays a dual role: in the absence of the ligand, P2X7R functions as a scavenger receptor involved in phagocytosis and following its activation by ATP, the P2X7R reduces proliferation of C cells (Genzen et al., 2009; Messemer et al., 2013; Leeson et al., 2018). It should be mentioned here that although these data appear contradictory with the fact that increased levels of extracellular nucleotides favor cell proliferation, ATP also acts on other receptors among which the metabotropic P2Y1R receptors that foster expansion of the C cell population (Suyama et al., 2012). The multiplicity of receptors targeted by extracellular nucleotides shapes a unique and selective response to these ligands.

#### TRPV (Transient Receptor Potential Vanilloid) Channels

Among the TRPV channels, TRPV1 has been detected within the V-SVZ in 20% of NSCs, in transient amplifying progenitors and in neuroblasts during the early postnatal period (Stock et al., 2014). TRPV1 expression then declines in 1 month old mice along with the diminution of neurogenesis, although radioautography figures published by Roberts et al. (2004) show some binding sites of TRPV1 in the V-SVZ of adult mice brain stained with radiolabeled TRPV1 ligands (Roberts et al., 2004; Stock et al., 2014). Interestingly, TRPV1 expression could be up-regulated in adulthood by physiological situations known to promote neurogenesis (Stock et al., 2014). In mice, deletion of TRPV1 conducted to a substantial rise in proliferating cells, but lesser differentiation to neurons or glia in the neurogenic niches of the postnatal brain. Thus, the loss of TRPV1 in neural stem/progenitor cells disturbs differentiation and the growth potential of V-SVZ stem cells, suggesting that TRPV1 coordinates the coupling between proliferation and differentiation of neural precursors (Stock et al., 2014).

### Intracellular Ca^2+^ Handling and Ca^2+^ Signaling Pathways

Several studies have underlined that maintenance of NSCs and of their activity requires an appropriate handling of the ER Ca^2+^ reservoir. For instance, the Ca^2+^ waves that promote NSC self-renewal following injury are IP3-dependent (Kraft et al., 2017). Conversely, activation of the Ca^2+^ efflux from the ER by the pro-apoptotic protein Bax increased cell death, which was substantially hampered by siRNA-mediated suppression of IP3R expression (Shi et al., 2005). These data underscore the necessity of a strict handling of Ca^2+^ for keeping the NSC population.

CaBP also play essential roles in modulation of NSC activity. Among these, NFAT, especially the NFATc1 and NFATc3 isoforms, is expressed in newborn rodent V-SVZ cell cultures (Serrano-Pérez et al., 2015). Interestingly, the recruitment of NFAT occurred only in response to local Ca^2+^ signals obtained following SOC activation but not global Ca^2+^ transients, illustrating that specific Ca^2+^ patterns are required for selective responses (Somasundaram et al., 2014). Analysis of NFAT function showed that the NFAT inhibitor VIVIT slowed cell cycle in NSCs and reduced their ability to differentiate (Serrano-Pérez et al., 2015). Yet, a possible involvement of NFAT for keeping a multipotential stem cell state, that has been observed in embryonic stem cells (MacDougall et al., 2019), remains to be investigated. Noteworthy, Ca^2+^ buffering proteins may also control stemness as it has been shown that S100B expression is associated with a loss of NSC potential of GFAP-expressing cells (Raponi et al., 2007).

In addition, a wide transcriptomic study in migrating neuroblasts isolated from the RMS identified the calmodulin signaling network as one of the four up-regulated networks in these cells (Khodosevich et al., 2009). Silencing *in vivo* the expression of specific genes of this network, namely calmodulin (calm1) and CamKinase (CamK4), conducted to a decrease in the numbers of neuroblasts that reached the olfactory bulb, indicating that calmodulin and its effector are essential players for neuroblast migration (Khodosevich et al., 2009).

## Physiopathological Roles of Ca^2+^ in GSCs

Alterations in the Ca^2+^ toolkit have been reported in human tissues resected from glioblastoma, with increased expressions of TRPC (C1, C6) and TRPV (V1, V2) channels as compared to normal tissue (Alptekin et al., 2015). Transcriptomic analysis of GSCs unveiled that Ca^2+^ channels and signaling pathways, that elicit vital cell functions in response to extracellular cues, are enriched in GSCs, as compared to more mature non-stem glioblastoma cells that express more Ca^2+^ buffers (Wee et al., 2014). Such alterations most likely lead to modified Ca^2+^ homeostasis and Ca^2+^ codes that subsequently, may trigger tumorigenic behavior. In accordance, the epigenetic drugs that increase stemness of GSCs modify the Ca^2+^ signaling pathway (Wang et al., 2018). Interestingly, a screen of a chemical library of 72 ion channel blockers identified that 10 drugs among the 12 drugs capable of reducing GSC viability act on Ca^2+^-related signaling networks, supporting that Ca^2+^ channels or transporters may be appealing targets in GSCs (Niklasson et al., 2017).

In the following sections, we will examine the involvement of the Ca^2+^ toolkit in GSCs by reviewing first the implication of plasma membrane channels and then, the function of intracellular Ca^2+^ handling and signaling pathways. An overview of the Ca^2+^ proteins expressed and their functions in GSCs is provided in Figure 4 and summarized in Table 1.

### Plasma Membrane Channels

#### Voltage-Gated Calcium Channels (VGCCs)

T-type voltage-gated Ca^2+^ channels (Ca_V_3.2) whose overexpression has been associated with a worse prognosis, have been found enriched in GSCs of glioblastoma as compared to either non-GSC-tumor cells or normal tissue (Wee et al., 2014; Zhang et al., 2017). Hypoxia, known to promote resistance to anticancer therapies, increased expression of the Ca_V_3.2 VGCC in GSCs. Conversely, treatment with mibefradil, a FDA-approved inhibitor of Ca_V_3.2 that is used to treat hypertension, substantially reduced the GSC population by promoting their differentiation and reducing their viability (Niklasson et al., 2017; Zhang et al., 2017). This effect was mimicked by RNAi-mediated attenuation of Ca_V_3.2 expression (Zhang et al., 2017). Because resistance to treatments represents a major hurdle in current oncology, the fact that oral administration of mibefradil in mice xenografted with GSCs sensitized them to temozolomide treatment and prolonged survival of mice, opens new perspectives to improve the therapies against glioblastoma (Zhang et al., 2017). Among the mechanisms that may underpin these effects, it has been proposed that T-Type blockers compromise GSC survival by blocking the Ca^2+^ influx required to recruit Ca^2+^-dependent K^+^ channels (KCa) whose activity is mandatory for the maintenance of cell polarity. This induces cell depolarization, thereby precluding the Na^+^-dependent transport of nutrients and ultimately leading to starvation (Niklasson et al., 2017).

#### Store-Operated Channels (SOCs)

SOCE is a ubiquitous Ca^2+^ influx that occurs in a wide range of cells, in response to numerous extracellular signals. During a characterization of the set of genes involved in Ca^2+^ signal generation differentially expressed in brain tumors and normal tissue, genes related to SOCE were found to be strongly perturbed, and one of the major actors of SOC, Orai 1, was disclosed as one of the genes whose expression is up-regulated in glioblastoma tissues and in GSCs (Robil et al., 2015). Already involved in promoting invasion capacities of glioblastoma cells (Motiani et al., 2013), Orai1 and/or SOC that are highly expressed in GSCs, could preserve or expand the population of GSCs. Indeed, inhibition of SOC channels with SKF-96365 reduced cell proliferation of GSCs and induced a quiescent transcriptomic signature in these cells (Aulestia et al., 2018). Altogether, these data suggest that SOCs may be pivotal for glioblastoma initiation, expansion, infiltration of the normal tissue and tumor relapse.

#### Ligand-Gated Ion Channels (LGICs)

As for NSCs, AMPA receptors have been detected at high concentrations in GSCs, as compared to the differentiated non-stem tumor cells (Oh et al., 2012; Wee et al., 2014). Two elegant studies recently established that AMPA receptors expressed by human glioma cells, cultured in stem cell conditions, mediate tumor cell interactions with surrounding neurons through authentic neuron-glioma glutamatergic synapses. It was shown that neuronal activity-mediated release of glutamate drives glioma progression by promoting glioma growth and invasion through Ca^2+^-dependent mechanisms involving AMPA receptors (Venkataramani et al., 2019; Venkatesh et al., 2019).

A screen of chemical libraries identified the nicotinic receptor antagonist, atracurium besylate, as a small molecule that effectively inhibits the clonogenic capacity and induces astroglial differentiation of patient-derived GSCs. Conversely, a nicotinic receptor agonist prevented atracurium besylate ability to reduce GSC self-renewal. Furthermore, this study showed that the survival of mice xenotransplanted with GSCs pretreated with atracurium besylate was significantly improved, suggesting that blockade of nicotinic cholinergic receptors may reduce GSC stemness and/or cell population (Spina et al., 2016).

Extracellular nucleotides found in the microenvironment also contribute to defining GSC activity. Indeed, it has been shown that as compared to more differentiated tumoral cells, GSCs release tenfold more extracellular adenosine (Torres et al., 2019) that may act as autocrine/paracrine ligand to activate G-protein-coupled P2Y1R purinergic receptors or ionotropic purinergic P2X7R receptors to respectively promote or restrain GSC proliferation (D’Alimonte et al., 2015).

#### TRPV Channels

Analysis of TRPV1 and TRPV2 expression showed that they are up-regulated in glioblastoma as compared to normal tissue (Alptekin et al., 2015). Studies of their possible functions demonstrated that the vanilloid receptor TRPV1 triggers tumor cell death in glioblastoma cells. Of interest it was shown that endovanilloids (TRPV1 ligands) are secreted by the NSCs that migrate to the tumor mass, and that the oncostatic effect that NSCs exert on the tumor involves the release of endovanilloids (Stock et al., 2014). TRPV2 on the other hand, reduces GSC stemness. Indeed, overexpression of TRPV2 was found to diminish GSC proliferation and promote their differentiation as glial cells both *in vitro* and *in vivo* in mice xenografted with TRPV2-overexpressing GSCs (Morelli et al., 2012).

### Intracellular Ca^2+^ Handling and Ca^2+^ Signaling Pathways

Ca^2+^ stores of intracellular organelles, the ER and mitochondria, seem also involved in modulating GSC properties, particularly in the acquisition of a quiescent phenotype, which allows GSCs to escape from current anti-cancer therapies. The involvement of IP3R has been found following the discovery of a selective cytotoxic agent called bisacodyl that was picked out during the screening of the chemical Prestwick library for its power of eradication of quiescent GSCs (Zeniou et al., 2015). Consecutive studies disclosed that this drug acts on IP3R to block Ca^2+^ efflux from the ER (Dong et al., 2017). Thus, the maintenance of the quiescence in GSCs and resistance to chemotherapies may rely on a specific handling of Ca^2+^ from the ER. In addition to ER, mitochondrial management of Ca^2+^ may be involved in the control of GSC stem cell state. In support of this hypothesis, transcriptomic analysis of the Ca^2+^ toolbox underlined an up-regulated expression of the mitochondrial Ca^2+^ transporter MCU and of the Ca^2+^/Na^+^ exchanger SLC8A3 in GSCs (Robil et al., 2015). Subsequently, it was described that proliferating cells have more sustained Ca^2+^ signals than quiescent GSCs and that these changes in Ca^2+^ responses are associated with mitochondrial remodeling, suggesting that mitochondrial Ca^2+^ might control quiescence of GSCs although this hypothesis needs to be experimentally challenged (Aulestia et al., 2018).

CaBP have also been involved in GSC stemness. Specifically, the levels of S100A4 expression have been identified as an independent prognostic indicator of glioma patient survival, the worse prognosis being associated with up-regulated S100A4 expression in patients with glioblastoma of the mesenchymal molecular subgroup (Chow et al., 2017). Further analysis showed that S100A4-expressing cells are enriched with GSCs and are required for GSC self-renewal and survival. Selective ablation of the S100A4-expressing cells in genetically engineered mice that form spontaneous gliomas was sufficient to compromise tumor growth (Chow et al., 2017).

Ca^2+^-dependent signaling pathways also contribute to GSC stemness or activity. Indeed, a recent study showed that NFAT1 expression is upregulated in GSCs as compared to more differentiated glioma cells. NFAT1 knockdown compromised GSC viability, impaired their self-renewal and migration abilities *in vitro*, and inhibited tumorigenesis *in vivo*. Conversely, NFAT1 overexpression promoted glioma growth through a mechanism involving the neurodevelopment protein 1-like 1 (NDEL1) that with NFAT1, controlled the maintenance of a naive stem cell state in GSCs (Jiang et al., 2019). In addition, NFAT2 may also represents a possible regulator of GSCs, especially of the mesenchymal glioblastoma subtype where it drives invasion and clonogenicity and promotes tumor growth through the regulation of HDAC1 (Song et al., 2020).

## Concluding Remarks

Our review highlights a central role of Ca^2+^ in both NSCs and GSCs, which is correlated to prominence of Ca^2+^-related transcripts in both cell types. Indeed, NSCs and GSCs possess, each, a specific Ca^2+^ toolkit that allows to maintain Ca^2+^ homeostasis and to encode specific Ca^2+^ signals in response to the numerous signals from their microenvironment. Several functions of specific Ca^2+^ components in NSCs and GSCs have been unveiled. Current available data underpins that stemness is controlled by VGCCs, SOCs and IP3Rs in NSCs while it is regulated by VGCCs, nicotinic receptors, TRPVs and NFAT in GSCs, indicating that there might be cell type specific effects of Ca^2+^ toolkit components. Such differences may rely on either a differential expression of Ca^2+^ toolkit elements that, in turn, generates differential responses, or on yet unexplored functions of specific Ca^2+^ components in GSCs and NSCs, or both. It should also be kept in mind that the Ca^2+^ signals produced in a cell not only depend on Ca^2+^ release in the cytoplasm but also on its extrusion, a process that influences the spatial and temporal extent of the Ca^2+^ signal. Concerning the pumps, whose activity shapes the Ca^2+^ responses, little is known on their expression and functions in either NSCs or GSCs.

*In vivo*, cells evolve in microenvironments with multiple extracellular cues, many of which elicit Ca^2+^ signals and control cellular activities. Thus, it will be of importance to understand how the different signals are integrated and conduct to a specific Ca^2+^ signal within each cell type. Along with this latter question, the downstream effectors recruited by the different Ca^2+^ signals will require further investigation in order to understand how the specific spatio-temporal and magnitude profiles of Ca^2+^ signals select a cellular response. Reciprocally, it will be essential to understand the impact of the microenvironment and of pathological states on the remodeling of the Ca^2+^ toolkit of NSCs and GSCs, as it has been observed for endothelial cells (Lodola et al., 2012; Moccia et al., 2020).

An in-depth knowledge of the Ca^2+^ toolkit in both NSCs and GSCs will contribute to understand the impact of Ca^2+^ alterations or dysregulation during aging and disease and will help to develop pharmacological strategies to combat brain diseases. Indeed, the remodeling of intracellular Ca^2+^ homeostasis is considered as a cellular and molecular hallmark of brain aging (Kumar et al., 2009; Gheorghe et al., 2014; Mattson and Arumugam, 2018) and represents one of the earliest abnormalities in both the familial and sporadic forms of Alzheimer’s disease (Alzheimer’s Association Calcium Hypothesis Workgroup, 2017; Popugaeva et al., 2018). However, age-related changes of the Ca^2+^ toolkit have not been explored in the V-SVZ so far. Yet, some molecules targeting the Ca^2+^ toolkit are already being tested in clinical trials for the treatment of glioblastoma. Among them, carboxyamidotriazole that inhibits non-VGCCs and disrupts Ca^2+^-mediated signal transduction, showed some promising results in a recent multicenter phase IB trial when used in combination with temozolomide, prompting further deciphering of the roles of Ca^2+^ channels in GSCs and NSCs (Omuro et al., 2018).

## Author Contributions

VC wrote the initial manuscript draft. VC, ET, ND, PA and BC revised and approved the manuscript. All authors contributed to the article and approved the submitted version.

## Conflict of Interest

The authors declare that the research was conducted in the absence of any commercial or financial relationships that could be construed as a potential conflict of interest.

## Abbreviations

AMPA: α-amino-3-hydroxy-5-méthylisoazol-4-propionate
CaBP: Ca^2+^ binding protein
CaMK: Ca^2+^/calmodulin-dependent protein kinase
CRAC: Ca^2+^ release activated Ca^2+^ channel
DAG: diacylglycerol
EGF: epidermal growth factor
ER: endoplasmic reticulum
GABA: gamma-aminobutyric acid
GFAP: glial fibrillary acidic protein
GPCR: G protein-coupled receptor
GSC: glioblastoma stem cell
IP3: inositol triphosphate
HDAC: histone deacetylase
IP3R: IP3 receptor
LGIC: ligand-gated ion channel
MCU: mitochondrial Ca^2+^ uniporter
nAChR: ionotropic cholinergic nicotinic receptors
NCX: Na^+^/Ca^2+^ exchanger
NFAT: nuclear factor activated T-cells
NMDA: N-methyl-D-aspartate
NSC: neural stem cell
NTDPase: nucleoside triphosphate diphosphohydrolase
P2X: P2Y receptors, purinergic receptors
PIP2: Phosphatidylinositol 4, 5-bisphosphate
PLC: phospholipase C
PMCA: plasma membrane Ca^2+^ ATPase
RCAS-TVA: replication-competent avian sarcoma-leukosis virus (RCAS) and its receptor the tumor virus A (TVA)
ROC: receptor-operated channel
ROCE: receptor-operated Ca^2+^ entry
RTK: receptor tyrosine kinases
RYR: ryanodine receptor
SDF1: stromal cell-derived factor 1
SERCA: sarco/endoplasmic reticulum Ca^2+^-ATPase
SOC: store-operated channel
SOCE: store-operated Ca^2+^ entry
STIM: stromal interaction molecule
TAP: transient amplifying progenitors
TRPC: transient receptor potential-canonical
TRPV: transient receptor potential vanilloid
VGCC: voltage-gated Ca^2+^ channels
V-SVZ: ventricular-subventricular zone.
